# In Vitro and In Silico Anthelmintic Activity of Extracts of *Lannea kerstingii* and *Ficus thonningii* on *Heligmosomoides polygyrus*

**DOI:** 10.1155/2024/1858154

**Published:** 2024-08-03

**Authors:** Ndjinkeu Ntcheuzing Serena, Masoud Besati, Noumedem Anangmo Christelle Nadia, Mahdi Yaghoobi, Yamssi Cédric, Claire Ciancia, Ngouyamsa Nsapkain Aboubakar Sidiki, Vincent Khan Payne, Mpoame Mbida, Haibo Hu

**Affiliations:** ^1^ Department of Animal Biology Faculty of Science University of Dschang, P.O. Box 067, Dschang, Cameroon; ^2^ Laboratory of Tropical and Emerging Infectious Diseases, Dschang, Cameroon; ^3^ Institute for Integrative Systems Biology (I2SysBio) CSIC−University of Valencia 46980, Paterna, Spain; ^4^ Department of Microbiology Haematology and Immunology Faculty of Medicine and Pharmaceutical Sciences University of Dschang, P.O. Box 96, Dschang, Cameroon; ^5^ Molecular Design and Synthesis Department of Chemistry KU Leuven, Celestijnenlaan 200F B-3001, Leuven, Belgium; ^6^ Department of Biomedical Sciences Faculty of Health Sciences University of Bamenda, P.O. Box 39, Bambili, Cameroon; ^7^ Wellcome Centre for Molecular Parasitology School for Infection and Immunity University of Glasgow, Glasgow, UK; ^8^ Jiangxi Province Key Laboratory of Pharmacology of Traditional Chinese Medicine National Engineering Research Center for Modernization of Traditional Chinese Medicine-Hakka Medical Resources Branch School of Pharmacy Gannan Medical University, Ganzhou 341000, China

**Keywords:** anthelmintics, *Ficus thonningii*, *Heligmosomoides polygyrus*, *Lannea kerstingii*

## Abstract

**Background:** The aim of this study was to assess the anthelmintic activity of *Lannea kerstingii* and *Ficus thonningii*, on a nematode model, to promote their use in the Cameroonian pharmacopoeia for the treatment of helminthiases.

**Methods:** One nematode was used, *Heligmosomoides polygyrus*. First, the effect of the extracts on the eggs and larval stages (L1, L2, and L3) of *H. polygyrus* was evaluated, 100 *μ*L of extract and 100 *μ*L of parasite suspension (containing 50 eggs) were mixed in a 96-well microplate. The 96-well microplate was incubated for 20 h at 25°C in the WMicroTracker which measures the motility of the worms at various concentrations. Finally, docking studies were conducted by using the Glide module in Schrodinger Maestro.

**Results:** The ethanolic extract of *L. kerstingii* with the half maximal inhibitory concentration (IC_50_) of 0.1371 mg/mL produced a higher ovicidal effect than the effect produced by other extracts of these plants. However, with an IC_50_ of 0.31 mg/mL, the aqueous extract of *F. thonningii* showed the greatest effect on the L2 stage. The aqueous and ethanolic extracts of *L. kerstingii* and *F. thonningii* inhibited the development of the L3 larvae of *H. polygyrus* with a better effect for the ethanolic extracts.

**Conclusion:** The use of *L. kerstingii* and *F. thonningii* for the treatment of helminthiasis has been proved in vitro and in silico by this research. However, more research is required, especially on the acute toxicity and in vivo anthelmintic efficacy to validate this scientific investigation.

## 1. Introduction

A class of diseases known as neglected tropical diseases (NTDs) predominantly occur in tropical areas [1]. Klohe et al. [2] report that of the 1.5 billion people globally who suffer from these diseases, 40% of them are from Africa. Pregnant or nursing mothers, as well as children of preschool or school age, are the most vulnerable individuals [3]. The most prevalent parasitic helminths that infect people are helminths such as *Ancylostoma duodenale*, *Necator americanus*, *Ascaris* spp., and *Trichuris* spp. Statistically one-sixth of all humans suffer from illnesses caused by these parasites [4].

There are roughly 20 NTDs and 12 of them are found in Cameroon, including helminthiases [5]. Animal and human parasite illnesses known as helminthiases are caused by helminth infection [6]. Moreover, another major issue facing the livestock business is helminth infections, which result in annual output losses exceeding billions of dollars [7]. The local populations use synthetic anthelmintics to fight these helminthiases; however, regular and careless use of synthetic anthelmintics reduces their efficacy by leading to helminth resistance [8]. We also observe that there is currently no vaccine to prevent these parasites from multiplying, that synthetic anthelmintics have harmful side effects, and that people in the region must pay a relatively high price for these medications [9]. This is particularly true for the livestock sector, where annual costs for recurrent pharmacological treatments in Britain alone approach one billion pounds sterling (£1000 million) [10]. One way to address these issues is to develop new strategies using medicinal herbs, which are readily accessible, comparatively less toxic, and less expensive [11, 12]. Thus, since more than two decades, the WHO promotes the development of novel plant-based treatments [13]. *Lannea kerstingii* Engl et K. Krause (Anacardiaceae), a deciduous tree which grows in the Noun division (Cameroon's Western region), has been studied and seems to have potential [14]. Indeed, it is used to treat bacterial infections [14, 15] and anemia [16] and as antitrypanosomiasis [17] and anticonvulsant [18]. Throughout Africa and beyond, a variety of fruit-bearing trees, including the common wild fig (*Ficus thonningii*), have long been utilized as medicinal remedies. Although *F. thonningii* is widely used in ethnomedical systems [19], *F. thonningii* has traditionally been administered to reduce inflammation and treat bacterial infections [20], treat malaria [21], and heal wound [15]. Traditional healers in Cameroon use the plants *L. kerstingii* and *F. thonningii* to treat intestinal worms. The stem and/or branches of *L. kerstingii* and *F. thonningii* are used as fuel wood and to make charcoal by the local community. A crucial tool in computer-assisted drug design and structural molecular biology is molecular docking. Predicting the main binding mode(s) of a ligand with a protein that has a known three-dimensional (3D) structure is the goal of ligand–protein docking [22]. The aim of this work was to assess the anthelmintic activity of *L. kerstingii* and *F. thonningii* to promote their use in the Cameroonian pharmacopoeia for the treatment of helminthiases, as there is currently no published data on the scientific validation of these medicinal plants as anthelmintic agents.

## 2. Materials and Methods

### 2.1. Collection and Identification of Plant Materials

Traditional healers in Cameroon use the stem barks of *L. kerstingii* and *F. thonningii* to treat intestinal worms. Stem barks of *L. kerstingii* and *F. thonningii* were collected in Machut and Massassa, villages situated in the Noun division (Western region of Cameroon). They were registered as *L. kerstingii* (39284/HNC) and *F. thonningii* (36710/HNC) at the National Herbarium of Cameroon in Yaounde. Identifications have been done by Dr. Eric Ngantsop using samples of these plants (bark, leaves, flowers, and fruits).

### 2.2. Preparation of Extracts

Since traditional practitioners prepared this herbal remedy during the survey using fermented palm wine (ethanol) or infusion, ethanol and water solvent were used for the extraction. After identification at the herbarium, stem barks were cut into small pieces and dried at room temperature under shade. Then, the bark was ground in a mill to obtain powder. The method described by Azizi et al. [23] was used to prepare the extracts. Briefly, 100 g of *L. kerstingii* and *F. thonningii* powders was weighed and each introduced into 1 L of 95% ethanol. The mixture was macerated for 72 h at room temperature. The homogenate obtained was filtered using Whatman paper. The filtrate obtained has been introduced into an oven at 40°C for evaporation and obtention of the ethanolic extract for 24 h. For the aqueous extract, 100 g of powder was introduced in 1 L of boiled water at 100°C and sealed until cooling. The homogenate was filtered using Whatman paper. The obtained filtrate was dried at 40°C for 48 h.

### 2.3. Isolation and Concentration of Embryonated Eggs of *Heligmosomoides polygyrus*

Fresh *H. polygyrus* eggs are concentrated by flotation method as reported by Cédric et al. [24]. Briefly, 2 g of fresh infected feces is mixed with a saturated NaCl solution, allowing parasite eggs to rise to the top. Eggs are collected from the surface of a glass slide. Then, distilled water was used to wash the slides and detach eggs. Three centrifugations at 1500 rpm for 10 mn were performed on the egg suspension to remove the salt solution and concentrate them. They were then incubated for 24 h at room temperature to become embryonated eggs for the hatching test.

### 2.4. Culture and Collection of *H. polygyrus* Larvae

*H. polygyrus* larvae were cultured using the method outlined by Johnston et al. [25]. Charcoal and feces were combined in a 1:1 ratio until the desired consistency was reached. On petri dishes with wet filter papers in the middle, the mixture was spread on a thin layer and incubated at 27°C for 48 h (for L1 larvae), 96 h (for L2 larvae), and 7 days (for L3 larvae). The wet filter paper was rinsed with distilled water several times to collect the larvae.

### 2.5. In Vitro Anthelminthic Activity Against *H. polygyrus*

The microtracker which measures the real-time mobility of the worms was used to measure the ovicidal and larvicidal activity in accordance with the method described by Cédric et al. [24]. To assess the extract's ovicidal efficacy, 50 embryonated *H. polygyrus* eggs were put in contact with various concentrations of extracts (0.078–2.5 mg/mL) in a 96-microplate. After 24-h incubation, larvae were tracked using the worm microtracker instrument to observe their movement on the plates at 27°C for 24 h.

The larvicidal activity against L1/L2/L3 H*. polygyrus* larvae was assessed using the same protocol with incubation in the worm microtracker for 24 h at 27°C.

The anthelminthic activity was then determined as follows [12]:

 $$\begin{array}{c} \text{Percentage of inhibition }\left(\%\text{inhibition}\right)=\frac{\text{mobility activity of control}-\text{mobility activity of the test sample}}{\text{mobility activity of control}}\times 100 \end{array}$$

### 2.6. Qualitative Phytochemical Screening and Determinations of Total Phenolic and Flavonoid Contents

The method described by Sidiki et al. [26] was used for the phytochemical screening and total phenolic and flavonoid content evaluation. Total phenolic and flavonoid content was determined as gallic acid equivalent (GAE).

### 2.7. Molecular Docking Study

**Figure 1:**
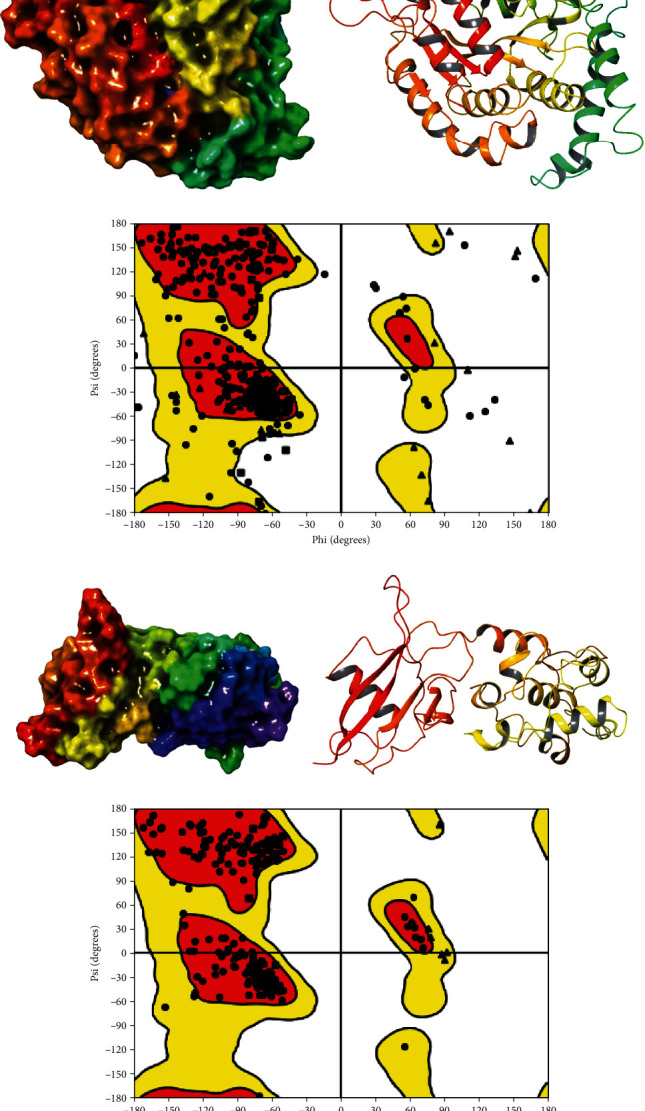
PDB ID: 1SA0. (a) The surface mode of the *β*-tubulin, (b) optimized *β*-tubulin receptor, and (c) Ramachandran plot of *β*-tubulin receptor. PDB ID: 4YSX. (d) The surface mode of the SDH model, (e) optimized SDH model receptor, and (f) Ramachandran plot of SDH model receptor.

Based on data found in various articles, particularly Khairuzzaman et al. [27] and Abongwa, Martin, and Robertson [28] showing that colchicine binding site of the *β*-tubulin protein target is a classic target of numerous anthelmintic substances, it has been chosen for this study. The crystal structure of the *β*-tubulin-colchicine complex (Protein Data Bank [PDB] ID: 1SA0) was provided in PDB format from the PDB website (https://www.rcsb.org/). Furthermore, several compounds derived from quinoline and triazole have been identified as potential inhibitors of succinate dehydrogenase (SDH) [29, 30]. The *β*-tubulin-colchicine complex utilized in this study was generated using the protein preparation wizard application of the Schrodinger software; it was then used for docking as the *β*-tubulin receptor. Acidic or basic amino acid residues were ionized in the appropriate state based on crystallographic water molecules without 3H bonds. Hydrogen bonds corresponding to pH 7.4 were then added as suitable ionization states were considered for both acidic and basic amino acids. Energy minimization of the crystal structure was achieved using OPLS3e force field [31, 32]. The compounds used in this research are constituents extracted from different parts of *L. kerstingii* [32–34] and *F. thonningii* [35, 36] and have been published in various articles in recent years. In this research, the purpose of the in silico part is to obtain information about the molecular interaction of ligands and proteins of nematodes in order to control them in detail. The docking was prepared with the described ligands. The key residues at the *β*-tubulin-colchicine binding site located in the *β*1 subunit were identified as Cys241, Leu248, Ala250, Leu255, Asn258, Met259, Ala316, Ala317, Val318, Val238, Lys352 Thr353, and Thr376 [27]. Glide, the receptor grid generation section of Maestro, was used to generate the grid boxes for *β*-tubulin as seen in Figure 1. By creating two boxes of dimensions 10 × 10 × 10 and 20 × 20 × 20, which represent the active site of *β*-tubulin, a grid center was calculated (*X*: 93.09, *Y*: 73.21, and *Z*: 012). Furthermore, SDH was chosen due to its high efficacy as an inhibitor against many nematode species, including *Caenorhabditis elegans* [30, 37]. In addition, certain quinoline and triazole compounds have been identified as potential inhibitors of SDH [29, 30]. The PDB does not include the crystal structure of *C. elegans* SDH. Therefore, we utilized the Swiss model web server to generate a 3D model of its structure. The subunit sequences of *C. elegans* (UniProt entry Q09545) were obtained from the UniProt database (http://uniprot.org). The most pertinent generated SDH structure was chosen based on the global model quality estimation (GMQE) and qualitative model energy analysis (QMEAN) values. The homology modelling technique was employed using SDH from *Ascaris suum* (PDB ID: 4YSX), which shares a sequence identity of 83.87% with the SDH of *C. elegans*. Due to the lack of a cocrystallized ligand with the *β*-tubulin chain and the SDH model developed, we utilized the Sitemap module of Schrodinger to identify probable cavities for inhibitor binding. We employed albendazole as a reference chemical to assess the strength of inhibition of tubulin polymerization. Albendazole is a widely used and commercially successful medicine that binds to the same pocket of tubulin [38]. Consequently, the tubulin structure was cleaned by removing water molecules and other substances such as ions, cofactors, and ligands. Hydrogens with polar characteristics were included, hydrogens with nonpolar characteristics were combined, and the process of histidine protonation was adjusted manually. The Kollman charges were computed for the entire enzyme structure and distributed among the individual residues. Further simulation required the creation of 3D structures for the compounds [39]. The 3D configuration of the reference ligands for SDH and tubulin-*β* was obtained from the PubChem portal (https://pubchem.ncbi.nlm.nih.gov). The receptor grid generation portion of Maestro, called Glide, was utilized to create the grid boxes for SDH. The active site of SDH was represented by two boxes with dimensions of 10 × 10 × 10 and 20 × 20 × 20. A grid center was then calculated at coordinates *X*, 93.95; *Y*, 21.69; and *Z*, 64.01.

Schrodinger Maestro's Glide module was used to conduct docking studies [40]. Possible adduct structures generated by molecular docking using the score function in the software have been ranked and grouped [41] (Tables 1 and 2). A 3D structure of any complex can be predicted based on the binding properties of ligands and targets. Prediction how ligands will appear within a specific binding site in terms of their conformation and orientation (or position) can be done using docking. “Protein Preparation wizard” was used in Maestro to preprocess the protein structure. The modules automatically generated state and refinement step phases that were used to bring hydrogen atoms and certain essential bonds to the missing protein molecule sites. As a result of the optimization process, the receptor grid generation was processed and docking scores with different forms of docked ligands were analyzed [42, 43].

### 2.8. Statistical Analysis

Microsoft Excel was used to determine the % inhibition based on the activity values. The concentration–response curves generated by plotting the logarithm of the concentration as a function of the % inhibition using the GraphPad Prism Version 8 software were then used to calculate the half maximal inhibitory concentrations (IC_50_). The docking studies were carried out using the Schrodinger Maestro software's Glide module. The software's score function was employed to categorize and rank different molecular docking-produced probable adduct structures.

## 3. Results

### 3.1. Effect of Extracts on Hatching

**Figure 2:**
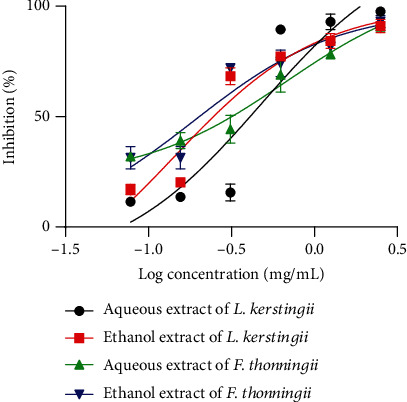
Inhibitory concentration 50 (IC_50_) of egg hatching treated with aqueous and ethanolic extracts of *L. kerstingii* and *F. thonningii.*

The ovicidal effect of aqueous and ethanolic extracts of *L. kerstingii* and *F. thonningii* on embryonated eggs of *H. polygyrus* is presented in Figure 2. As expected, albendazole (positive control) produced a total effect (100%) on inhibiting the hatching of embryonated eggs and the negative control (distilled water) induced no effect. Furthermore, *L. kerstingii* or *F. thonningii* extracts produced concentration-dependent ovicidal (% inhibition) effects. Thus, IC_50_ of 0.47 and 0.14 mg/mL corresponds, respectively, to those of the aqueous and ethanolic extracts of *L. kerstingii* and the IC_50_'s of 0.69 and 0.19 mg/mL correspond, respectively, to the aqueous and ethanolic extracts of *F. thonningii.* In both cases, the ethanolic extracts showed greater ovicidal effects compared to the aqueous extracts. However, the ethanolic extract of *L. kerstingii* with an IC_50_ of 0.1371 mg/mL produced a higher ovicidal effect than all other extracts. The detailed results of these ovicidal activities are presented as Table S1.

### 3.2. Effect of Extracts on *H. polygyrus* L1 Larvae

**Figure 3:**
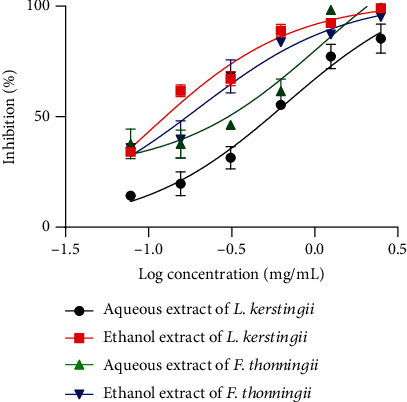
First larval stage and larval motility IC_50_ of *H. polygyrus* when treated with aqueous and ethanolic extracts of *L. kerstingii* and *F. thonningii.*

Figure 3 illustrates the effects of aqueous and ethanolic extracts of *L. kerstingii* and *F. thonningii* on L1 larvae of *H. polygyrus.* Albendazole (positive control) caused death of all L1 larvae, and the negative control (distilled water) had no effect on this larval stage. Extracts of *L. kerstingii* and *F. thonningii* had concentration-dependent larvicidal effects on L1 larvae. Therefore, the IC_50_'s of the aqueous and ethanolic extracts of *L. kerstingii* were 0.67 and 0.10 mg/mL, while the IC_50_ of *F. thonningii* was 1 and 0.19 mg/mL for the aqueous and ethanolic extracts, respectively. Compared to aqueous extracts, ethanolic extract had a more significant effect on the development of *H. polygyrus* L1 larvae. However, the larvicidal effect of the ethanolic extract of *L. kerstingii* on L1 was higher than the effect produced by other extracts. The detailed results of these larvicidal activities are presented as Table S2.

### 3.3. Effect of Extracts on *H. polygyrus* L2 Larvae

**Figure 4:**
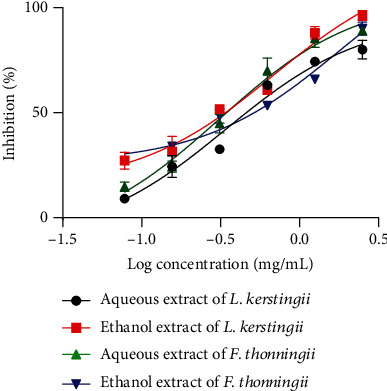
Second larval stage and larval motility IC_50_ of *H. polygyrus* when treated with aqueous and ethanolic extracts of *L. kerstingii* and *F. thonningii.*

Figure 4 shows the effects of aqueous and ethanolic extracts of *L. kerstingii* and *F. thonningii* on L2 larvae of *H. polygyrus*. Albendazole had 100% mortality on L2 larvae, while the negative control (distilled water) had no effect. Compared to the ethanolic extracts, the aqueous extracts of the two plants had a higher inhibition on the development of L2 larvae of *H. polygyrus.* However, a greater effect was observed with the aqueous extract of *F. thonningii*, with a low IC_50_ of 0.31 mg/mL. The detailed results of these larvicidal activities are presented as Table S3.

### 3.4. Effect of Extracts on *H. polygyrus* L3 Larvae

**Figure 5:**
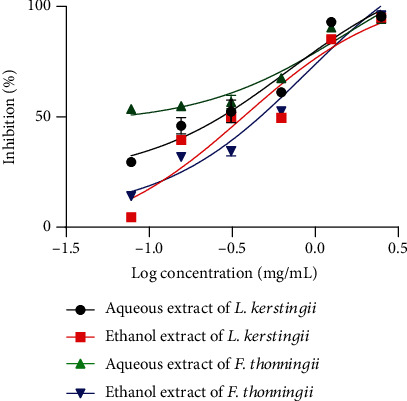
Third larval stage and larval motility IC_50_ of *H. polygyrus* when treated with aqueous and ethanolic extracts of *L. kerstingii* and *F. thonningii.*

The larvicidal effect of the aqueous and ethanolic extract of *L. kerstingii* and *F. thonningii* on the L3 larvae of *H. polygyrus* is presented in Figure 5. The aqueous and ethanolic extracts of *L. kerstingii* and *F. thonningii* inhibited the development of the L3 larva of *H. polygyrus* with a better effect for the ethanolic extracts. The ethanolic extract of *L. kerstingii* with an IC_50_ of 0.39 mg/mL produced a better larvicidal effect on L3 compared to other extracts. The detailed results of these larvicidal activities are presented as Table S4.

Table 3 shows the phytochemical screening of the aqueous and ethanol extracts of *L. kerstingii* and *F. thonningii*.

It follows from the analysis of this table that all extracts do not contain quinones. Similarly, the ethanolic extracts of *L. kerstingii* did not contain alkaloids.

Table 4 shows the quantity of flavonoids and polyphenols present in each extract. Ethanolic extracts contain more flavonoids than aqueous extracts. Similarly, more polyphenolic compounds were found in the ethanol extract of *L. kerstingii* compared to the aqueous extract (713.70 ± 5.88 vs. 440.43 ± 18.72).

### 3.5. Analysis of Molecular Docking and In Silico Approaches for Anthelminthics

Using the Glide module, molecular docking between ligands and target proteins was performed [44, 45]. Several ligands demonstrated significant docking scores when they interacted with amino acids in target proteins. An overview of docking scores for the five leading ligands is provided in Tables 1 and 2.

HTVS, SP, and XP molecular docking methodologies were used to screen compounds from *L. kerstingii* and *F. thonningii*. A sample of 15% of the most stable ligands was screened in every step based on their docking scores. The most stable structures of ligands were docked using the XP docking score.

**Figure 6:**
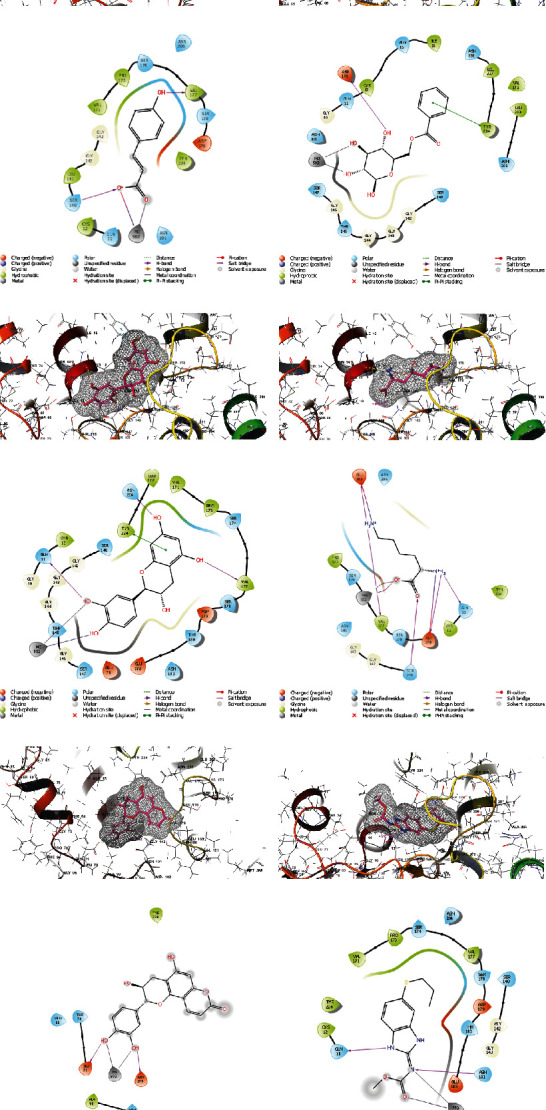
Three-dimensional (3D) and two-dimensional (2D) interactions between the *β*-tubulin and the ligands of *L. kerstingii*: (a) p-coumaric acid, (b) vacciniin, (c) cianidanol, (d) 2-O-caffeoylglucarate, (e) phyllocoumarin, and (f) albendazole.

In Figure 6, the five compounds' docking scores and binding interactions with the *β*-tubulin receptor are compared. In *L. kerstingii*, p-coumaric acid interacts most with Cys12, Val171, Pro173, and Leu141, because of its hydrophobicity, and its carbonyl oxygen interacts most with MG502. Its hydroxyl group interacts with hydrophobic Val177, and the charged oxygen interacts with positive charged Ser140 which is why it shows the highest inhibitory rate compared to other compounds. Similar to p-coumaric acid, vacciniin binds to the receptor and inhibits *β*-tubulin due to its flavonoid backbone and hydroxyl group interaction with MG502 and Asp179. The ring of this compound interacts as Pi-Pi stacking with Tyr224.

Cianidanol binds to the receptor with hydroxyl groups as h-bond donor to Asn206, Val177, Gln11, and MG502. The ring of cianidanol, like vacciniin, interacts as Pi-Pi stacking with Tyr224.

2-O-Caffeoylglucarate is a hydrogen donor to Glu183, Val177, Asp179, and Gln11. The carbonyl group of 2-O-caffeoylglucarate gets hydrogen from Ser140. The ammonium groups of this ligand either have salt bridges with Glu183 and Asp179. Phyllocoumarin is the fifth compound which interacts with *β*-tubulin, because of interactions of hydroxyl groups of structures with Glu71, Asp179, and MG502 amino acids of *β*-tubulin receptor.

According to docking calculations, four compounds p-coumaric acid, vacciniin, cianidanol, and 2-O-caffeoylglucarate had a stronger interaction than the positive control (albendazole) with *β*-tubulin receptor.

**Figure 7:**
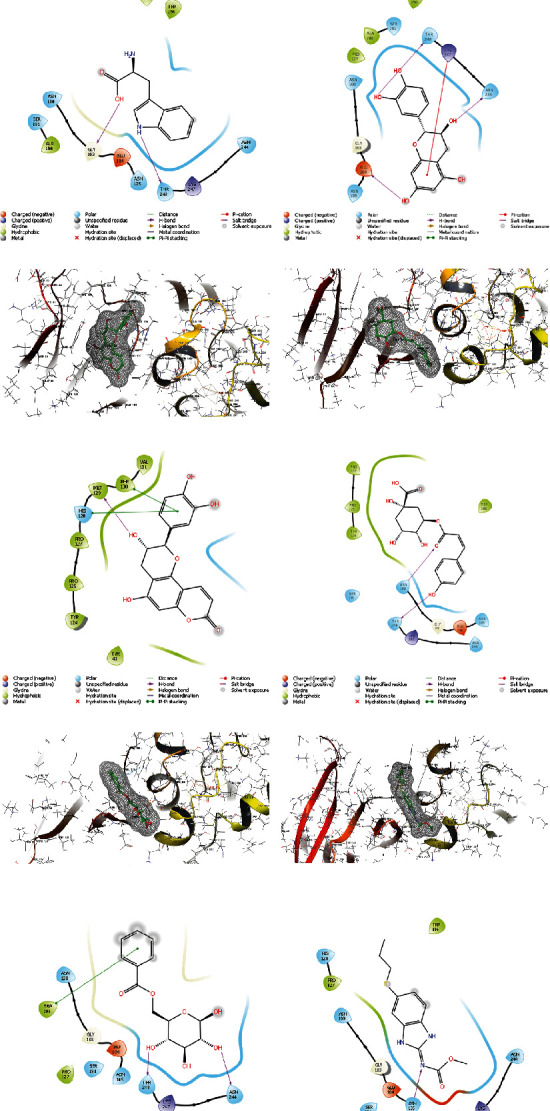
Three-dimensional (3D) and two-dimensional (2D) interactions between the SDH and the ligands of *L. kerstingii*: (a) L-tryptophane, (b) cianidanol, (c) phyllocoumarin, (d) 5-p-coumaroylquinic acid, (e) vacciniin, and (f) albendazole.

In Figure 7, the five compounds' docking scores and binding interactions with the SDH receptor are presented. In *L. kerstingii*, L-tryptophane interacts most with Thr248 and Gly103, because of its hydrogen donor interactions. Cianidanol binds to the receptor and inhibits SDH due to its flavonoid backbone and hydroxyl group interaction with Glu104 Thr248, and the ring of this compound interacts as Pi-Pi stacking with Lys247. Phyllocoumarin binds to receptor with hydroxyl group as h-bond donor to Met129 and Pi-Pi stacking of their ring to Phe130 and His128. 5-p-Coumaroylquinic acid is a hydrogen donor to Thr248 and hydrogen acceptor from Asn100. The hydrophobicity interactions are significant. Vacciniin is the fifth compound which interacts with SDH, because of interactions of hydroxyl groups of structures with Thr248 and Asn244 amino acids of receptor.

According to docking calculations, all of the five compounds, L-tryptophane, cianidanol, phyllocoumarin, vacciniin, and 5-p-coumaroylquinic acid, had a stronger interaction than the positive control (albendazole) with SDH receptor.

**Figure 8:**
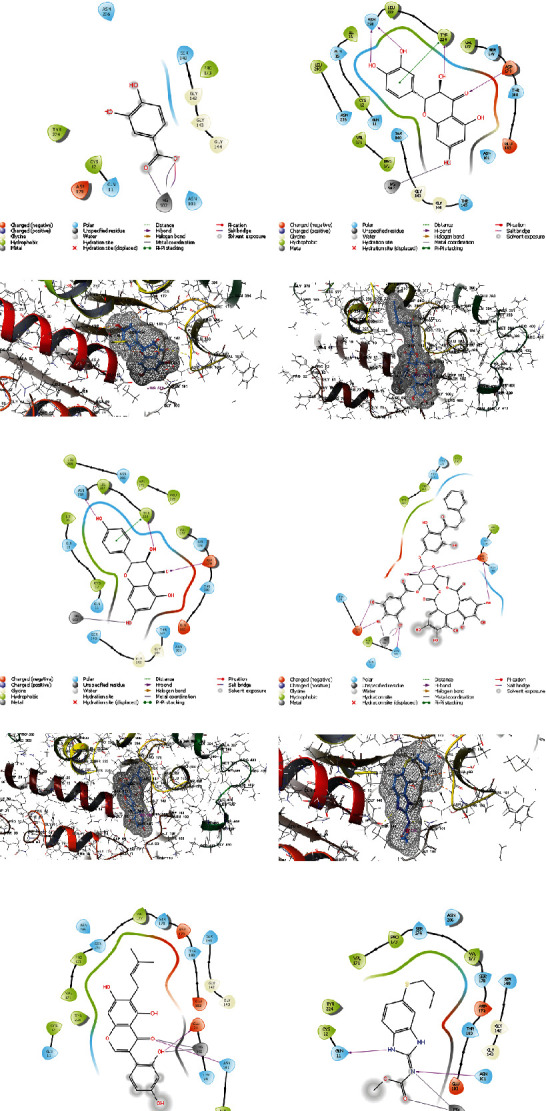
Three-dimensional (3D) and two-dimensional (2D) interactions between the *β*-tubulin and ligands of *F. thonningii*: (a) protocatechuic acid, (b) dihydroquercetin, (c) dihydrokaempferol, (d) thonningianin A, (e) luteone, and (f) albendazole.

In Figure 8, the five compounds' docking scores and binding interactions with the *β*-tubulin receptor are compared. In *F. thonningii*, protocatechuic acid is a major metabolite of anthocyanin that exhibits its greatest inhibitory effects via polar section of receptor's site and the salt bridge of charged oxygen and metal coordination with MG502.

As a flavonoid, dihydroquercetin is the second inhibitory compound of *F. thonningii* due to its interactions with the receptors Asn228, Tyr224, MG502, and Asp179. Its inhibitory effect is caused by the hydroxyl groups attached to the amino acids. Likewise, dihydrokaempferol is a flavonoid and, like dihydroquercetin, its inhibitory effect is caused by the hydroxyl groups attached to the amino acids, Asn228, Tyr224, Asp179, and MG502. In the presence of the connection between its hydroxyls and the receptor, thonningianin A inhibits *β*-tubulin because of the effects of giving and receiving the H of its hydroxyls. And luteone like other components interacts with *β*-tubulin with its connections between hydroxyl and carbonyl groups with Glu71, Asn101, and MG502.

According to docking calculations, three compounds protocatechuic acid, dihydroquercetin, dihydrokaempferol had a stronger interaction than the positive control (albendazole) with *β*-tubulin receptor.

**Figure 9:**
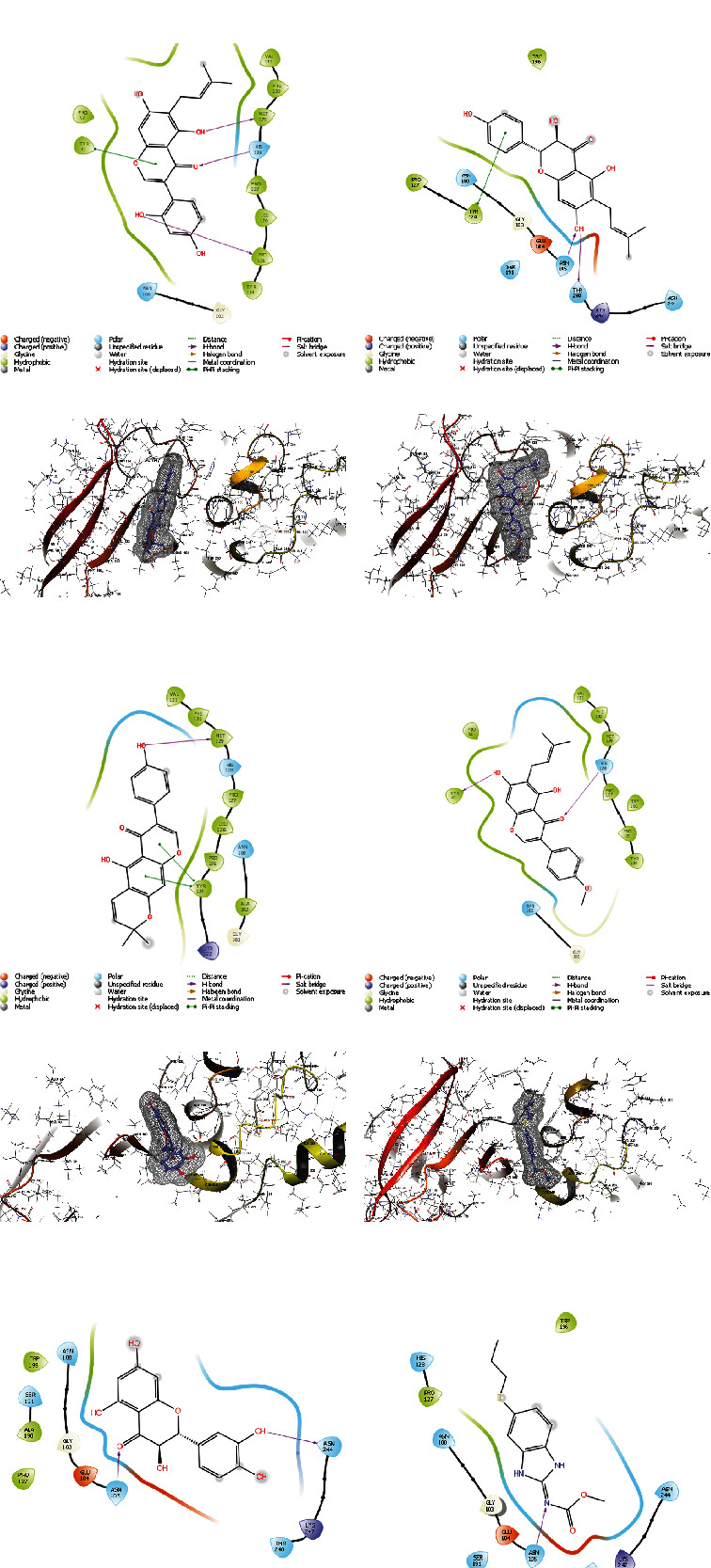
Three-dimensional (3D) and two-dimensional (2D) interactions between the SDH and the ligands of *F. thonningii*: (a) luteone, (b) shuterin, (c) alpinumisoflavone, (d) gancaonin A, (e) taxifolin, and (f) albendazole.

In Figure 9, the five compounds' docking scores and binding interactions with the SDH receptor are presented. In *F. thonningii*, luteone is a major metabolite of a prenylated isoflavone that exhibits its greatest inhibitory effects via polar section of receptor's site and Pi-Pi stacking with Tyr41 and hydrogen donor and acceptors with Met129, Pro125, and His128. As a flavonoid, shuterin is the second inhibitory compound of *F. thonningii* due to its interactions with the receptors Asn105, Thr248, and Tyr124. Its inhibitory effect is caused by the hydroxyl groups attached to the amino acids and Pi-Pi stacking interaction. Likewise, alpinumisoflavone is a flavonoid, and its inhibitory effect is caused by the hydroxyl group attached to the Met129 and Pi-Pi stackings with Tyr124. Gancaonin A inhibits SDH because of the effects of giving and receiving the H with His128 and Tyr41. And taxifolin like other components interacts with SDH with its connections between hydroxyl and carbonyl groups with Asn244 and Asn105.

According to docking calculations, all of the five compounds of luteone, shuterin, alpinumisoflavone, gancaonin A, and taxifolin had a stronger interaction than the positive control (albendazole) with SDH receptor.

## 4. Discussion

The IC_50_'s of *H. polygyrus* eggs hatched in the aqueous and ethanolic extract of *L. kerstingii* were 0.47 and 0.14 mg/mL, respectively. Those of *F. thonningii* were 0.6784 and 0.1783 mg/mL for the aqueous and ethanolic extracts, respectively. From these results, the two plant extracts are considered very active with concentration-dependent effects. Increasing the concentration of plant extracts results in an increase in the inhibition rate. Moreover, the ethanolic extracts inhibited egg hatching more. This observation is in agreement with that observed by Koné et al. [46] on the activity of medicinal plants used in the North of Côte d'Ivoire against intestinal helminths and those obtained by Njinga et al. [47] on the evaluation of the antidiarrheal effect of *L. kerstingii.* This ovicidal effect could be justified by the content of bioovicidal compounds present in the extracts [24]. Compounds such as flavonoids, alkaloids, saponins, triterpenes, tannins, and polyphenols were much more present in these ethanolic extracts, thus favoring the passage of secondary metabolites inside the cell. They interfere with the mechanism of cellular mitosis by paralyzing the larva present in the eggs in the case of embryonated eggs [48]. Furthermore, when the larvae seek to absorb water from the surrounding environment to swell and break the membrane to escape, they find that the latter contains molecules harmful to their survival and therefore find themselves trapped and end up dying [49]. All these plant extracts had a larvicidal effect on the L1, L2, and L3 larvae of *H. polygyrus* with a better effect on the L1, L3, and L4 stage larvae of the ethanolic extracts, while on the stage L2, it is the aqueous extracts which had a better effect. These results are similar to those obtained by Dube et al. [50] on the anthelmintic and cytotoxic activity of compounds isolated from the fruits of *Ozoroa insignis*. The chemical composition of the extracts of *L. kerstingii* and *F. thonningii* revealed the presence of flavonoids, phenols, tannins, saponins, alkaloids, and triterpenes. On the other hand, quinones were absent. Indeed, numerous studies have revealed the importance of these metabolites in the fight against helminths. In particular, flavonoids whose activity would consist of blocking the phosphorylation reaction, thus inhibiting energy production in parasitic worms, which would lead to their death [51]; saponins which exert their anthelmintic activity by inhibiting acetylcholinesterase, which causes paralysis of the worms and their death [52]; terpenes exhibit anthelmintic activities causing intestinal damage to the parasite [53]; tannins would help kill nematodes by interfering with the absorption of nutrients by the worms from the host cell, or when the condensed tannins are ingested by the larvae, they would bind to the intestinal mucosa of the parasitic worms and thus cause the autolysis [54]; alkaloids showed anthelmintic activity by targeting acetylcholine receptors and suppressing glucose absorption, which resulted in the death of helminths [55]. In addition, the observation of the presence of these secondary compounds is in agreement with the observations of H. Usman, Abdulrahman, and A. Usman [56] which shows that the barks of *F. thonningii* contain these secondary compounds.

This larvicidal effect could be justified by the fact that after the penetration of bioactive substances (secondary metabolites) into the cuticle, they can act on the larvae at several levels: First, they can prevent the absorption of glucose or block post receptors, thus paralyzing the larvae; then, they can also induce the release of gamma aminobutyric acid (GABA) which blocks the transmission of nerve impulses or uncouple the oxidative phosphorylation reaction which can lead to the urging of energy from the larvae; finally, they can bind to free proteins available in the gastrointestinal tract of the larvae causing anorexia and ultimately death of the worm [57]. It is obvious that ethanol solvent extracts substances from plants better than water. Indeed, this could be due to the fact that the extraction of plant products varies depending on the solvent used, which is in agreement with the results of Marie-Magdeleine et al. [58] who showed that the biological activity of plant extracts depends on the type of solvent used. We evaluated some natural compounds within the active extracts analyzed in the past literature from *L. kerstingii* [32–34] and *F. thonningii* [35, 36] which may bind to the colchicine binding site of the *β*-tubulin and SDH subunits thereby contributing to the observed anthelmintic effects. A library comprising phytochemicals reported from *L. kerstingii* and *F. thonningii* was employed for virtual screening targeted at the colchicine binding site of the *β*-tubulin subunit. The two natural compounds p-coumaric acid (from *L. kerstingii*) and protocatechuic acid (from *F. thonningii*) exhibited potent binding affinity to the target protein, and their interactions mostly relied on h-bond acceptor and donor interactions. The p-coumaric acid and protocatechuic acid emerged as the best-scoring ligands with a binding affinity of −8.766, and −8.571 kcal/mol for molecules, respectively. p-Coumaric acid formed hydrogen bonds with three key amino acid residues of the target protein, namely, Ser140, Val177, and MG502, along with hydrophobic and polar interactions with more key amino acid residues. Protocatechuic acid formed salt bridge in hydrophobic section of receptor with MG502 and has polar interaction with amino acid residues of the target protein, namely, Ser140, Pro173, and Asn206, along with hydrophobic and polar interactions with more key amino acid residues. This finding is in agreement with previous report [27, 59]. According to the calculation of the interactions between the ligands of *F. thonningii* and *L. kerstingii* with *β*-tubulin protein receptor, the ligands rich in hydroxyl groups interact with amino acids more effectively. Phytochemical and flavonoid compounds in these two plants are largely responsible for their anthelminthic action.

We were unable to do the phytochemical analyses (composition) of the different parts (leaves, roots, fruits, etc.) of the plants and to identify the different ligands that might have been present in our extract by performing an HPLC fingerprint on the extracts due to our limited resources.

## 5. Conclusion

On the eggs, L1, L2, and L3 of *H. polygyrus*, the extracts of *L. kerstingii* and *F. thonningii*, showed anthelmintic activity. Additional in vivo anthelminthic and toxicity testing is needed to scientifically validate their usage by the local population. In silico comparative studies of these compounds on the *β*-tubulin-colchicine complex and SDH protein receptors and their interactions with amino acids confirm that these compounds have effective effects on the receptor protein due to the hydroxyl and amine groups and flavonoid backbones of most of these compounds.

## Figures and Tables

**Table 1:** Docking scores of ligands of *L. kerstingii* with *β*-tubulin and SDH.

**No.**	**Compounds**	**Docking scores of *β*-tubulin**	**No.**	**Compounds**	**Docking scores of SDH**
1	p-Coumaric acid	−8.766	1	L-Tryptophane	−6.011
2	Vacciniin	−7.398	2	Cianidanol	−5.883
3	Cianidanol	−6.711	3	Phyllocoumarin	−5.606
4	2-O-Caffeoylglucarate	−6.448	4	5-p-Coumaroylquinic acid	−5.138
5	Phyllocoumarin	−5.729	5	Vacciniin	−4.513
Positive control	Albendazole	−6.151	Positive control	Albendazole	−4.257

**Table 2:** Docking scores of ligands of *F. thonningii* with *β*-tubulin and SDH.

**No.**	**Compounds**	**Docking scores of *β*-tubulin**	**No.**	**Compounds**	**Docking scores of SDH**
1	Protocatechuic acid	−8.571	1	Luteone	−6.413
2	Dihydroquercetin	−6.650	2	Shuterin	−6.049
3	Dihydrokaempferol	−6.621	3	Alpinumisoflavone	−6.039
4	Thonningianin A	−6.088	4	Gancaonin A	−5.678
5	Luteone	−6.026	5	Taxifolin	−5.581
Positive control	Albendazole	−6.151	Positive control	Albendazole	−4.257

**Table 3:** Qualitative phytochemical screening.

**Test**	**Plant extracts**			
	***F. thonningii***		***L. kerstingii***	
	**Aqueous**	**Ethanolic**	**Aqueous**	**Ethanolic**
Alkaloids	**+**	**+**	**+**	**−**
Phenols	**+**	**+**	**+**	**+**
Tannin	**+**	**+**	**+**	**+**
Quinones	**−**	**−**	**−**	**−**
Saponins	**+**	**+**	**+**	**+**
Flavonoids	**+**	**+**	**+**	**+**
Triterpenes	**+**	**+**	**+**	**+**

**Table 4:** Total phenolic and flavonoid contents.

**Plants**	**Extracts**	**Metabolites**	
		**Polyphenols**	**Flavonoids**
*L. kerstingii*	Aqueous	440.43 ± 18.72^a^	302.5 ± 24.04^a^
	Ethanolic	713.70 ± 5.88^b^	359.2 ± 15.41^a^
*F. thonningii*	Aqueous	657.63 ± 12.39^c^	435.83 ± 35.39^a^
	Ethanolic	530.12 ± 4.01^d^	446.70 ± 35.21^a^

*Note:* The results are presented as mean ± standard deviation. Values with the same superscript letter in the same column are not significant with different letters (*p* < 0.05).

## Data Availability

All data generated and analyzed are included in this research article.

## Supporting Information

Supporting Information Additional supporting information can be found online in the Supporting Information section. Supporting Table S1: Percentage of inhibition of hatching of embryonated eggs of *Heligmosomoides polygyrus* and IC_50_ of aqueous and ethanolic extracts. Table S2: Percentage of inhibition of L1 larvae of *Heligmosomoides polygyrus* and IC_50_ of aqueous and ethanolic extracts of *L. kerstingii* and *F. thonningii*. Table S3: Percentage of inhibition of L2 larvae of *Heligmosomoides polygyrus* and IC_50_ of aqueous and ethanolic extracts of *L. kerstingii* and *F. thonningii*. Table S4: Percentage of inhibition of L2 larvae of *Heligmosomoides polygyrus* and IC_50_ of aqueous and ethanolic extracts of *L. kerstingii* and *F. thonningii*.
