# Supplementary material for: In Vitro and In Silico Anthelmintic Activity of Extracts of Lannea kerstingii and Ficus thonningii on Heligmosomoides polygyrus
Source: J Parasitol Res. 2024 Aug 3;2024:1858154. doi: 10.1155/2024/1858154 (PMC11316912; doi:10.1155/2024/1858154)
Supplement: Supporting Information — Additional supporting information can be found online in the Supporting Information section. Supporting Table S1: Percentage of inhibition of hatching of embryonated eggs of Heligmosomoides polygyrus and IC50 of aqueous and ethanolic extracts. Table S2: Percentage of inhibition of L1 larvae of Heligmosomoides polygyrus and IC50 of aqueous and ethanolic extracts of L. kerstingii and F. thonningii. Table S3: Percentage of inhibition of L2 larvae of Heligmosomoides polygyrus and IC50 of aqueous and ethanolic extracts of L. kerstingii and F. thonningii. Table S4: Percentage of inhibition of L2 larvae of Heligmosomoides polygyrus and IC50 of aqueous and ethanolic extracts of L. kerstingii and F. thonningii. [file 1858154.f1.docx]

**Table S1: Percentage of inhibition of hatching of embryonated eggs of *Heligmosomoides polygyrus* and IC50 of aqueous and ethanolic extracts of *L. kerstingii* and *F. thonningii***

| Plants | Extracts | Concentrations (mg/mL) | | | | | | IC_50_  (mg/mL) | Albendazole  5µg/ml | Negative  control |
| --- | --- | --- | --- | --- | --- | --- | --- | --- | --- | --- |
|  |  | **0.078** | **0.156** | **0.312** | **0.625** | **1.25** | **2.5** | 0.47 | 100 | 0 |
| *L. kerstingii* | Aqueous | 11.46±0.16^a^ | 13.61±0.99^a^ | 15.66±3.88^a^ | 89.47±1.77^b^ | 92.94±3.49^b^ | 97.66±1.24^b^ |  |  |  |
|  | Ethanolic | 16.95±2.36^a^ | 20.23±1.46^a^ | 68.29±3.84^b^ | 77.24±0.3^b^ | 84.28±3.37^b^ | 91.08±3.03^b^ |  |  |  |
| *F. thonningii* | Aqueous | 31.95±0.51^a^ | 39.13±3.61^a^ | 44.31±6.36^a^ | 68.96±7.85^a^ | 78.22±1.38^b^ | 90.71±0.39^b^ | 0.14 | 100 | 0 |
|  | Ethanolic | 31.38±5.01^a^ | 31.38±5.01^a^ | 71.97±0.26^b^ | 74.16±6.07^b^ | 81.68±0.4^b^ | 93.01±2.81^b^ | 0.69 | 100 | 0 |

The results are presented as Mean ± Standard Deviation. a, b, c, d values with the same superscript letter in the same column are not significant with different letters (P˂0.05).

**Table S2: Percentage of inhibition of L1 larvae of *Heligmosomoides polygyrus* and IC_50_ of aqueous and ethanolic extracts of *L. kerstingii* and** ***F. thonningii***

| Plants | Extracts | Concentrations (mg/mL) | | | | | | IC_50_  (mg/mL) | Albendazole  5µg/ml | Negative  control |
| --- | --- | --- | --- | --- | --- | --- | --- | --- | --- | --- |
|  |  | **0.078** | **0.156** | **0.312** | **0.625** | **1.25** | **2.5** | 0.69 | 100 | 0 |
| *L. kerstingii* | Aqueous | 14.30±1.18^a^ | 19.69±5.36^a^ | 31.46±5.01^a^ | 55.36±1.56^b^ | 77.29±5.46^b^ | 85.41±6.66^b^ |  |  |  |
|  | Ethanolic | 34.21±1.81^a^ | 61.83±2.72^a^ | 67.18±3.17^a^ | 88.98±2.88^b^ | 92.48±2.01^b^ | 99.05±0.28^b^ | 0.10 | 100 | 0 |
| *F. thonningii* | Aqueous | 34.68±4.23^a^ | 37.74±6.30^a^ | 46.29±1.29^a^ | 61.51±5.51^a^ | 98.38±0.31^b^ | 99.47±0.09^b^ | 1 | 100 | 0 |
|  | Ethanolic | 35.88±0.18^a^ | 39.67±8.42^a^ | 68.32±7.52^b^ | 83.72±0.17^b^ | 87.21±0.28^b^ | 95.10±0.85^b^ | 0.19 | 100 | 0 |

The results are presented as Mean ± Standard Deviation. a, b, c, d values with the same superscript letter in the same column are not significant with different letters (P˂0.05).

| Plants | Extracts | Concentrations (mg/mL) | | | | | | IC_50_  (mg/mL) | Albendazole  5µg/ml | Negative  control |
| --- | --- | --- | --- | --- | --- | --- | --- | --- | --- | --- |
|  |  | **0.078** | **0.156** | **0.312** | **0.625** | **1.25** | **2.5** | 0.34 | 100 | 0 |
| *L. kerstingii* | Aqueous | 9.15±0.32^a^ | 24.48±5.19^a^ | 32.60±0.65^a^ | 63.19±0.77^b^ | 74.39±0.67^b^ | 80.03±4.45^b^ |  |  |  |
|  | Ethanolic | 27.20±4^a^ | 31.50±7.22^a^ | 51.70±0.51^b^ | 60.69±0.3^b^ | 88.08±2.99^c^ | 96.30±2.34^c^ | 0.68 | 100 | 0 |
| *F. thonningii* | Aqueous | 14.82±2.21^a^ | 24.35±2.61^a^ | 44.84±4.41^a^ | 69.97±6.12^b^ | 85.34±4.26^b^ | 89.01±0.74^b^ | 0.31 | 100 | 0 |
|  | Ethanolic | 27.31±0.15^a^ | 34.01±0.26^a^ | 48.42±3.35^b^ | 53.59±0.33^b^ | 65.85±1.14^b^ | 90.08±3.11^b^ | 1.73 | 100 | 0 |

**Table S3: Percentage inhibition of L2 larvae of *Heligmosomoides polygyrus* and IC_50_ of aqueous and ethanolic extracts of *L. kerstingii* and**

***F. thonningii***

The results are presented as Mean ± Standard Deviation. a, b, c, d values with the same superscript letter in the same column are not significant with different letters (P˂0.05).

**Table S4: Percentage inhibition of L2 larvae of *Heligmosomoides polygyrus* and IC_50_ of aqueous and ethanolic extracts of *L. kerstingii* and *F. thonningii***

| Plants | Extracts | Concentrations (mg/mL) | | | | | | IC_50_  (mg/mL) | Albendazole  5µg/ml | Negative  control |
| --- | --- | --- | --- | --- | --- | --- | --- | --- | --- | --- |
|  |  | **0.078** | **0.156** | **0.312** | **0.625** | **1.25** | **2.5** | 0.70 | 100 | 0 |
| *L. kerstingii* | Aqueous | 29.50±0.20^a^ | 45.93±3.70^b^ | 52.46±5.12^b^ | 61.11±1.59^b^ | 92.94±0.39^c^ | 95.42±0.62^c^ |  |  |  |
|  | Ethanolic | 4.5±0.2^a^ | 39.50±0.2^b^ | 50±2.04^b^ | 49.50±0.2^b^ | 85.16±0.41^c^ | 94.91±1.1^c^ | 0.39 | 100 | 0 |
| *F. thonningii* | Aqueous | 53.50±0.65^a^ | 54.88±0.43^a^ | 56.67±3.12^a^ | 67.50±1.02^b^ | 90.42±0.17^c^ | 94.40±0.62^c^ | 1.48 | 100 | 0 |
|  | Ethanolic | 14.16±0.47^a^ | 31.86±1.28^b^ | 34.45±2.12^b^ | 52.50±1.02^c^ | 91.34±1.04^d^ | 95.93±0^d^ | 0.89 | 100 | 0 |

The results are presented as Mean ± Standard Deviation. a, b, c, d values with the same superscript letter in the same column are not significant with different letters (P˂0.05).
